# Lymphovascular Invasion is an Independent Negative Prognostic Factor in Esophageal Adenocarcinoma

**DOI:** 10.1245/s10434-024-15717-8

**Published:** 2024-07-17

**Authors:** Britton B. Donato, Megan E. Campany, Justin T. Brady, J. Asher Jenkins, Richard Butterfield, Valerie Armstrong, Staci E. Beamer, Pedro Reck dos Santos, Jonathan D’Cunha

**Affiliations:** 1 Department of Cardiothoracic Surgery, Mayo Clinic, Phoenix, AZ USA; 2 Division of Cardiothoracic Surgery, Medical College of Wisconsin, Milwaukee, WI USA; 3 Mayo Clinic Alix School of Medicine, Mayo Clinic, Scottsdale, AZ USA; 4 Department of Surgery, Mayo Clinic, Phoenix, AZ USA; 5 Department of Quantitative Health Sciences, Mayo Clinic, Phoenix, AZ USA

**Keywords:** Esophageal adenocarcinoma, Lymphovascular invasion, Nodal upstaging, Survival

## Abstract

**Background:**

The significance of lymphovascular invasion (LVI) in esophageal adenocarcinoma (EAC) has not yet been described. Potential utility as an adjunct to current staging guidelines remains unknown.

**Methods:**

The National Cancer Database was queried from 2006 to 2020. Univariate and multivariable models, Kaplan Meier method, and log-rank test were used. Subgroup analyses by pN stage were conducted.

**Results:**

Of 9,689 patients, 23.2% had LVI. LVI was an independent prognostic factor (hazard ratio [HR] 1.401, 95% confidence interval [CI] 1.307–1.502, *p* < 0.0001) with reduction in median survival to 20.0 months (95% CI 18.9–21.4) from 39.7 months (95% CI 37.8–42.3, *p* < 0.0001). Multivariable survival analysis adjusted on pN and pT stage found that patients with LVI had decreased survival in a given pN stage (*p* < 0.001). pN0/LVI+ patients had a similar prognosis to the higher staged pN1/LVI− (28.6 months), although pN1/LVI− patients did slightly worse (*p* = 0.0135). Additionally, patients with pN1/LVI+ had equivalent survival compared with pN2/LVI− (*p* = 0.178) as did pN2/LVI+ patients compared with pN3/LVI− (*p* = 0.995).

**Conclusions:**

In these data, LVI is an independent negative prognostic factor in EAC. LVI was associated with a survival reduction similar to an upstaged nodal status irrespective of T stage. Patients with LVI may be better classified at a higher pN stage.

Esophageal cancer is the sixth leading cause of cancer-related death worldwide with a poor prognosis despite evolving multimodal treatment. Esophageal adenocarcinoma (EAC) is the most common histologic subtype in developed countries and is increasing in prevalence in contrast to the global decrease in esophageal squamous cell carcinoma (ESCC).^1^ Current clinical staging relies on esophagogastroduodenoscopy (EGD) with biopsy, esophageal ultrasound (EUS) with fine-needle aspiration (FNA), and PET/CT imaging. Despite these advancements in staging modalities, accurate prognostication from clinical staging remains limited.^2^ As such, identification of additional prognostic factors to guide patient counseling, adjuvant therapy, and postoperative surveillance is essential.

The presence of lymphovascular invasion (LVI), defined as the presence of tumor cells within an endothelial lining, has been implicated as a poor prognostic factor in both the rate of complete pathologic response (CPR) and overall survival trends in a variety of cancers. While the pathophysiologic mechanism of this process has yet to be conclusively detailed, it is thought that LVI is an immediate precursor to lymph node involvement, thus explaining the associated reduction in survival.^3^ Specific to esophageal cancer, studies of LVI in ESCC have implicated LVI as a prognostic factor independent of TNM stage.^4,5^ Recent demonstration of the feasibility of pathologic nodal (pN) upstaging in ESCC patients with LVI further underscores the importance of acknowledging LVI in prognostication.^6^

While such data exist for ESCC, there remains a paucity of data characterizing the association of LVI with EAC patient outcomes. Thus, using a large national database, we examined the utility of LVI as an independent prognostic factor in EAC. Additionally, as LVI is thought to provide the foundation for nodal involvement, we evaluated the impact of LVI on survival within a given pN stage.

## Materials and Methods

The National Cancer Database (NCDB) was queried for patients with EAC diagnosed between 2006 and 2020. Patients were included in the analysis if they had been diagnosed with nonmetastatic EAC and had complete survival data. Only those that received neoadjuvant chemoradiation and underwent esophagectomy were included to comply with the standard-of-care established by the CROSS trial. Those that were coded according to the AJCC 6th and 7th edition staging guidelines were restaged to match eighth edition standards by using appropriate TNM staging criteria. Comparisons between those with LVI and those without LVI were made by using chi-square and equal-variance *t*-test where appropriate. Univariate and multivariable logistic regression analysis was used to identify characteristics associated with LVI and positive margins. Univariate analyses of survival and pN/LVI subgroup comparisons were conducted by using the Kaplan-Meier method. Univariate and multivariable Cox proportional hazard models were used to evaluate LVI as an independent predictor of overall survival. Multivariable logistic and hazard models were adjusted on univariately significant predictors. All analyses were two-sided and considered statistically significant at the *p* = 0.05 level. Analyses were performed in SAS v9.4 (SAS Institute, Cary, NC). This study was submitted to the institutional review board (IRB) at Mayo Clinic Hospital and deemed exempt.

## Results

**Fig. 1:**
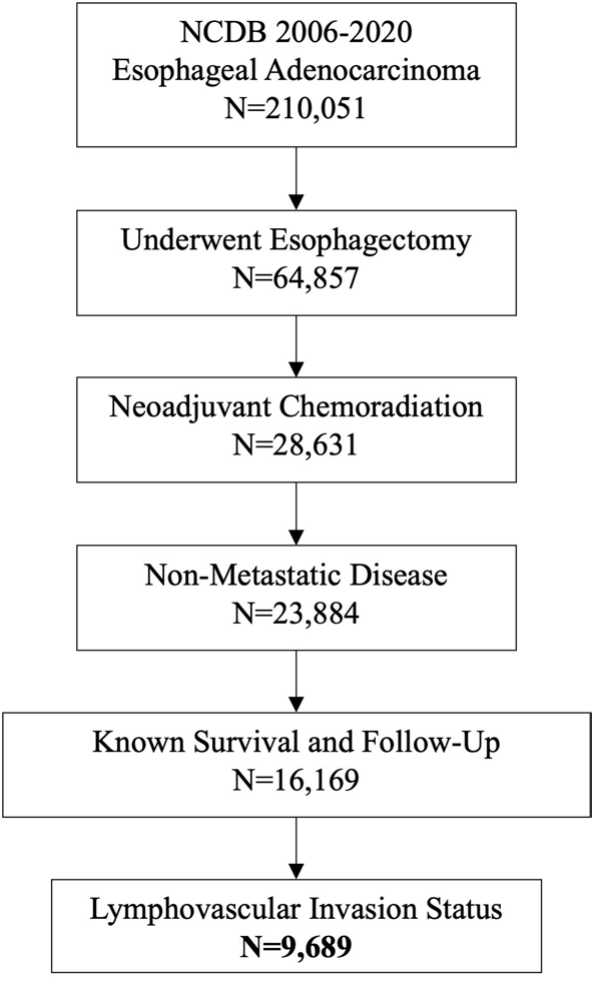
Consort diagram

We identified 9,689 patients who met study criteria, of whom 23.2% (2250) were LVI+ (Fig. 1). The samples were largely comparable from a sociodemographic perspective with patients tending to be White (93.6%), male (82.5%), and in their mid-60s at the time of diagnosis (median age 64 years, IQR 57–69 years). The majority of patients were diagnosed with clinical stage III disease (81.3%). A higher proportion of LVI was observed in male patients (23.6% vs. 21.2% of females, *p* = 0.049), in those who received immunotherapy (36.0% vs. 23.0% without immunotherapy, *p* < 0.0001), and in clinical stage III disease (24.5% vs. 16.9% stage II disease, *p* < 0.001; Table 1).

**Table 1:** Demographic and clinical characteristics

	LVI Absent *N* = 7,439	LVI Present *N* = 2,250	Total *N* = 9,689	p-value
*Age at diagnosis (years)*				**0.0060**^1^
Mean (SD)	63.0 (9.1)	62.4 (9.3)	62.9 (9.2)	
Median (IQR)	64 (57–69)	63 (56–69)	64 (57–69)	
Range	23.0–89.0	22.0–88.0	22.0–89.0	
*Sex*				**0.0494**^2^
Male	6,309 (76.4%)	1,946 (23.6%)	8,255 (85.2%)	
Female	1,130 (78.8%)	304 (21.2%)	1,434 (14.8%)	
*Race*				0.4341^2^
White	6,946 (76.6%)	2,119 (23.4%)	9,065 (93.6%)	
Black	267 (78.8%)	72 (21.2%)	339 (3.5%)	
American Indian	21 (77.8%)	6 (22.2%)	27 (0.3%)	
Asian	63 (85.1%)	11 (14.9%)	74 (0.8%)	
Other	142 (77.2%)	42 (22.8%)	184 (1.9%)	
*Ethnicity*				0.9078^2^
Non-Hispanic	7,083 (76.8%)	2,144 (23.2%)	9,227 (97.1%)	
Hispanic	215 (77.1%)	64 (22.9%)	279 (2.9%)	
*Insurance Payor*				0.3887^2^
Uninsured	126 (74.6%)	43 (25.4%)	169 (1.8%)	
Medicare/medicaid	3,857 (77.3%)	1,130 (22.7%)	4,987 (52.1%)	
Private	3,376 (76.3%)	1,048 (23.7%)	4,424 (46.2%)	
*Facility treatment type*				**0.0122**^2^
Academic/research	3,616 (75.7%)	1,163 (25.5%)	4,779 (50.0%)	
Community CA	273 (78.4%)	75 (21.6%)	348 (3.6%)	
Comp. community	2,042 (79.0%)	544 (21.0%)	2,586 (27.0%)	
Integrated network	1,415 (76.5%)	436 (23.6%)	1,851 (19.4%)	
*Year of diagnosis*				**< 0.0001**^2^
2006–2011	1,075 (80.9%)	253 (19.1%)	1,328 (13.7%)	
2012–2015	2,972 (78.1%)	835 (21.9%)	3,807 (39.3%)	
2016–2020	3,392 (74.5%)	1,162 (25.5%)	4,554 (47.0%)	
*Diagnosis to surgery (days)*				**0.0034**^1^
Mean (SD)	143.2 (41.9)	146.3 (50.3)	143.9 (44.0)	
Median (IQR)	135 (118–158)	136 (118–162)	135 (118–159)	
Range	7.0–637.0	25.0–921.0	7.0–921.0	
*Clinical stage*				**< 0.0001**^1^
I	588 (86.7%)	90 (13.3%)	678 (7.3%)	
II	887 (83.1%)	181 (16.9%)	1,068 (11.4%)	
III	5,738 (75.5%)	1,864 (24.5%)	7,602 (81.3%)	
*Pathologic stage*				
0	1,041 (94.6%)	60 (5.4%)	1,101 (12.2%)	
I	1,640 (93.9%)	107 (6.1%)	1,747 (19.4%)	
II	1,033 (84.0%)	197 (16.0%)	1,230 (13.7%)	
III	3,101 (64.4%)	1,713 (35.6%)	4,814 (53.5%)	
IV	29 (25.9%)	83 (74.1%)	112 (1.2%)	
*Surgical margins*				
Positive	347 (50.4%)	342 (49.4%)	689 (7.2%)	
Negative	7,031 (78.8%)	1,887 (21.2%)	8,918 (92.8%)	

Bold values indicate statistically significant at *p* < 0.05

^1^Equal variance two sample t-test

^2^Chi-Square p-value

On univariate analysis, race (*p* = 0.45), ethnicity (*p* = 0.91), median income (*p* = 0.79), education level (*p* = 0.43), and insurance payor (*p* = 0.39) did not impact the probability of LVI. On multivariable analysis sex (*p* = 0.091) did not impact probability of LVI, whereas older age (by a 1-year increase) was found to be associated with a lesser probability of LVI (odds ratio [OR] 0.993, 95% confidence interval [CI] 0.987–0.999, *p* = 0.016). Treatment facility type also was associated with LVI (comprehensive community cancer program OR 0.846, 95% CI 0.751–0.954, *p* = 0.04). Not surprisingly, clinical stage was found to be a strongly associated with LVI (clinical stage II OR 1.354, 95% CI 1.028–1.784, *p* < 0.0001) (clinical stage III OR 2.144, 95% CI 1.703–2.698, *p* < 0.0001; Table 2).

**Table 2:** Logistic regression analysis of lymphovascular invasion

	Univariate analysis		Multivariable analysis	
	OR (95% CI)	p-value	OR (95% CI)	p-value
*Age: one year increase*	0.993 (0.988–0.998)	**0.0060**	0.993 (0.987–0.999)	**0.0163**
*Diagnosis to surgery: one day increase*	1.002 (1.00–1.003)	**0.0036**	1.002 (1.001–1.003)	**0.0007**
*Sex*				
Male	Reference	**0.0495**	Reference	0.0910
Female	0.873 (0.761–1)		0.885 (0.767–1.02)	
*Race*				
White	Reference	0.4473		
Black	0.884 (0.678–1.152)			
Asian	0.574 (0.302–1.09)			
American Indian	0.937 (0.378–2.323)			
Other	0.97 (0.685–1.373)			
*Ethnicity*				
Non-Hispanic	Reference	0.9082		
Hispanic	0.983 (0.741–1.305)			
*Median income (by zip code)*				
>$63,333	Reference	0.7907		
$50,354–$63,332	1.068 (0.936–1.219)			
$40,227–$50,353	1.043 (0.91–1.195)			
$<40,227	1.046 (0.892–1.226)			
*High school degree (by zip code)*				
<6.3%	Reference	0.4299		
6.3–10.8%	0.964 (0.842–1.103)			
10.9–17.5%	1.055 (0.919–1.211)			
>17.6%	0.931 (0.787–1.103)			
*Insurance Payor*				
Private Insurance	Reference	0.3888		
Medicare/Medicaid	0.944 (0.857–1.039)			
Uninsured	1.099 (0.772–1.565)			
*Facility treatment type*				
Academic/research	Reference	**0.0123**	Reference	**0.0395**
Community cancer	0.854 (0.656–1.112)		0.848 (0.647–1.113)	
Comp. community	0.828 (0.738–0.93)		0.846 (0.751–0.954)	
Integrated network	0.958 (0.845–1.087)		0.969 (0.85–1.103)	
*Clinical stage*				
Stage I	Reference	**< 0.0001**	Reference	**< 0.0001**
Stage II	1.333 (1.014–1.752)		1.354 (1.028–1.784)	
Stage III	2.122 (1.69–2.666)		2.144 (1.703–2.698)	

Bold values indicate statistically significant at *p* < 0.05

**Fig. 2:**
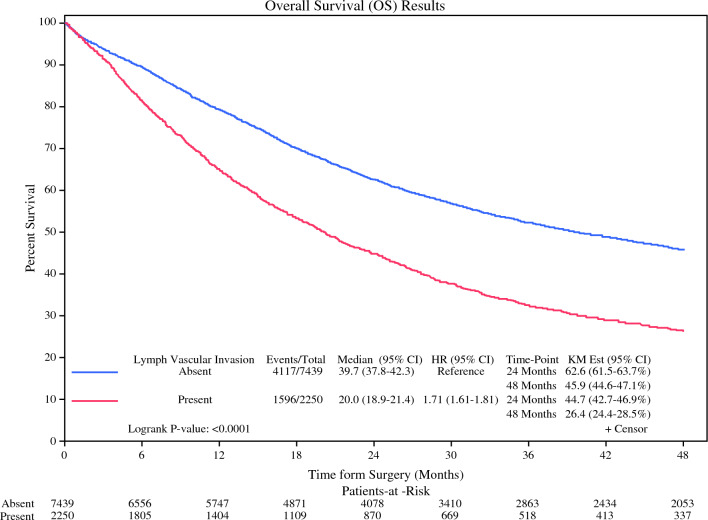
Kaplan–Meier curve depicting overall survival in allcomers with and without lymphovascular invasion

We evaluated the impact of LVI on survival in this population. Across all stages, patients with LVI had a nearly 50% reduction in median survival (20 months, 95% CI 18.9–21.4 vs. 39.7 months, 95% CI 37.8–42.3, *p* < 0.0001). Four-year survival was reduced from 45.9% (95% CI 44.6–47.1) to 26.4% (95% CI 24.4–28.5, *p* < 0.0001) in those that were LVI+ (Fig. 2). On multivariable analysis of overall survival adjusting for univariately significant demographic and clinical characteristics, including pathologic stage, LVI was found to be a strong independent prognostic factor (HR 1.401, 95% CI 1.307–1.502, *p* < 0.0001; Table 3).

**Table 3:** Logistic regression model of residual tumor margins

	Univariate analysis		Multivariate analysis	
	OR (95% CI)	p-value	OR (95% CI)	p-value
*LVI*				
Absent	Reference	**< 0.0001**	Reference	**< 0.0001**
Present	3.297 (2.815–3.862)		1.925 (1.604–2.31)	
*Age: one year increase*	0.99 (0.981–0.998)	**0.0131**	0.995 (0.985–1.004)	0.2553
*Diagnosis to surgery: one day increase*	1.003 (1.002–1.005)	**< 0.0001**	1.004 (1.002–1.005)	**< 0.0001**
*Sex*				
Male	Reference	0.7535		
Female	1.036 (0.83–1.294)			
*Race*				
White	Reference	0.1063		
Black	1.597 (1.095–2.331)			
Asian	0.544 (0.17–1.735)			
American Indian	1.606 (0.479–5.381)			
Other	0.955 (0.527–1.73)			
*Ethnicity*				
Non-Hispanic	Reference	0.8798		
Hispanic	0.964 (0.6–1.55)			
*Median income (by zip code)*				
>$63,333	Reference	0.1717		
$50,354–$63,332	1.195 (0.961–1.487)			
$40,227–$50,353	1.239 (0.992–1.548)			
$< 40,227	1.238 (0.956–1.604)			
*High school degree (by zip code)*				
< 6.3%	Reference	0.1092		
6.3–10.8%	1.299 (1.04–1.622)			
10.9–17.5%	1.299 (1.04–1.622)			
>17.6%	1.223 (0.93–1.609)			
*Insurance Payor*				
Private insurance	Reference	0.8689		
Medicare/medicaid	1.024 (0.874–1.2)			
Uninsured	1.148 (0.655–2.011)			
*Facility treatment type*				
Academic/research	Reference	**0.0080**	Reference	**0.0050**
Community cancer	1.648 (1.138–2.387)		1.646 (1.109–2.442)	
Comp. community	1.287 (1.07–1.549)		1.35 (1.111–1.641)	
Integrated network	1.138 (0.92–1.408)		1.159 (0.929–1.446)	
*pT stage*				
pT1	Reference	**< 0.0001**	Reference	**< 0.0001**
pT2	1.315 (0.952–1.816)		1.144 (0.824–1.588)	
pT3	4.094 (3.165–5.296)		2.666 (2.033–3.496)	
pT4	14.341 (8.553–24.047)		7.002 (4.04–12.137)	
*pN stage*				
pN0	Reference	**< 0.0001**	Reference	**< 0.0001**
pN1	1.672 (1.369–2.041)		1.207 (0.976–1.494)	
pN2	3.085 (2.491–3.821)		1.679 (1.319–2.136)	
pN3	5.967 (4.615–7.738)		2.905 (2.173–3.883)	

Bold values indicate statistically significant at *p* < 0.05

**Fig. 3:**
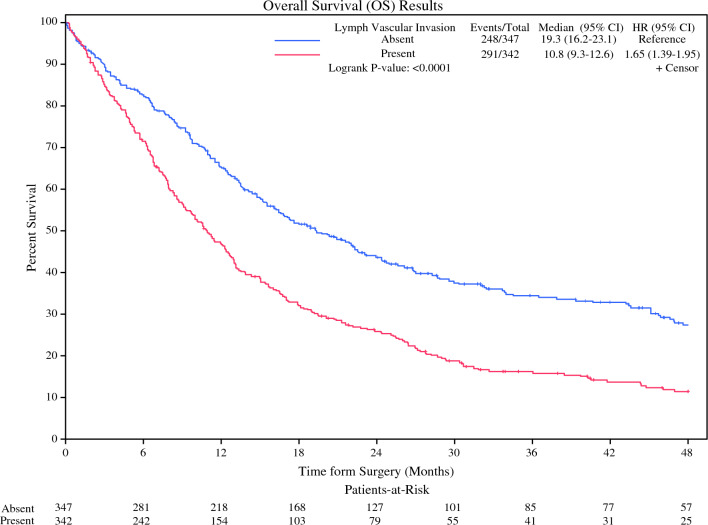
Kaplan–Meier curve depicting overall survival in those with residual margins and lymphovascular invasion compared to those with residual tumor margins without lymphovascular invasion

On a separate logistic regression model evaluating positive margins following resection, the presence of LVI was associated with a positive margin (OR 1.925, 95% CI 1.604–2.31, *p* < 0.0001). Treatment facility type also was associated with residual margin positivity (community cancer program OR 1.646, 95% CI 1.109–2.442, *p* = 0.005) (comprehensive community cancer program OR 1.35, 95% CI 1.111–1.641, *p* = 0.005; Table 4). The presence of positive residual margins conveyed an increased hazard of death (HR 1.466, 95% CI 1.323–1.626, *p* < 0.0001). The reduction in survival associated with LVI persisted among those with residual tumor margins; those with positive margins and LVI demonstrated a median survival of 10.8 months (95% CI 9.3–12.6) compared with 19.3 months (95% CI 16.2–23.1, *p* < 0.0001) in those with positive margins that did not have LVI (Fig. 3).

**Table 4:** Cox proportional hazard analysis of overall survival

	Univariate analysis		Multivariable analysis	
	HR (95% CI)	p-value	HR (95% CI)	p-value
*LVI*				
Absent	Reference	**< 0.0001**	Reference	**< 0.0001**
Present	1.709 (1.613–1.812)		1.401 (1.307–1.502)	
*Age: one year increase*	1.012 (1.009–1.015)	**< 0.0001**	1.013 (1.009–1.017)	**< 0.0001**
*Diagnosis to surgery: one day increase*	1.001 (1.0001–1.002)	**< 0.0001**	1.002 (1.001–1.003)	**< 0.0001**
*Era of diagnosis*				
2006–2011	Reference	**0.0003**	Reference	**< 0.0001**
2012–2015	0.927 (0.861–0.998)		0.91 (0.837–0.989)	
2016–2020	0.857 (0.793–0.926)		0.812 (0.742–0.889)	
*Sex*				
Male	Reference	**< 0.0001**	Reference	**0.0003**
Female	0.815 (0.754–0.88)		0.85 (0.779–0.927)	
*Race*				
White	Reference	0.2569		
Black	1.036 (0.899–1.195)			
Asian	1.128 (0.839–1.518)			
American Indian	1.057 (0.647–1.726)			
Other	0.799 (0.646–0.987)			
*Ethnicity*				
Non-Hispanic	Reference	0.0572		
Hispanic	0.847 (0.713–1.005)			
*Median income (by zip code)*				
>$63,333	Reference	**0.0001**	Reference	0.0585
$50,354–$63,332	1.078 (1.002–1.159)		1.027 (0.945–1.116)	
$40,227–$50,353	1.09 (1.011–1.175)		1.039 (0.947–1.14)	
$< 40,227	1.217 (1.117–1.326)		1.164 (1.039–1.303)	
*High school degree (by zip code)*				
< 6.3%	Reference	**0.0193**	Reference	0.8207
6.3–10.8%	1.047 (0.972–1.127)		1.035 (0.952–1.124)	
10.9–17.5%	1.116 (1.033–1.204)		1.043 (0.947–1.149)	
>17.6%	1.115 (1.018–1.221)		1.025 (0.91–1.156)	
*Insurance Payor*				
Private insurance	Reference	**< 0.0001**	Reference	**0.0006**
Medicare/medicaid	1.22 (1.157–1.286)		1.102 (1.027–1.183)	
Uninsured	1.412 (1.169–1.704)		1.414 (1.134–1.762)	
*Facility treatment type*				
Academic/research	Reference	**0.0008**	Reference	**0.0001**
Community cancer	1.201 (1.046–1.379)		1.096 (0.936–1.283)	
Comp. community	1.107 (1.041–1.178)		1.152 (1.074–1.236)	
Integrated network	1.096 (1.023–1.175)		1.149 (1.061–1.243)	
*Pathologic stage*				
Stage 0	Reference	**< 0.0001**	Reference	**< 0.0001**
Stage I	1.065 (0.945–1.199)		1.042 (0.913–1.188)	
Stage II	1.506 (1.331–1.704)		1.49 (1.3–1.708)	
Stage III	2.085 (1.878–2.314)		1.939 (1.724–2.181)	
Stage IV	3.299 (2.553–4.262)		2.875 (2.161–3.825)	

Bold values indicate statistically significant at *p* < 0.05

**Fig. 4:**
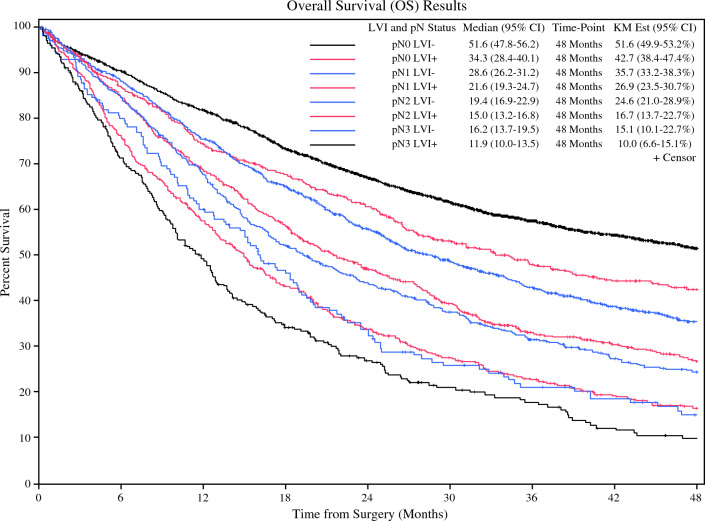
Kaplan–Meier curve depicting overall survival by pN stage and LVI status

Multivariable survival analysis of LVI adjusted for pathologic tumor (pT) stage showed that patients with LVI had significantly decreased survival compared with those without LVI in a given pN stage (*p* < 0.001). pN0/LVI+ patients had significantly worse median survival than pN0/LVI− patients (34.3 months, 95% CI 28.4–40.1 months vs. 51.6 months, 95% CI 47.8–56.2 months, *p* < 0.0001). pN0/LVI+ patient survival was closer to higher staged pN1/LVI− patients (respectively, 34.3 months, 95% CI 28.4–40.1 months vs. 28.6 months, 95% CI 26.2–31.2 months, HR 1.197, 95% CI 1.038–1.38, *p* = 0.0135). When evaluating median survival in those with LVI compared with the next highest nodal stage, similar trends were observed. No significant difference in median survival was found between pN1/LVI+ patients and pN2/LVI− patients (21.6 months, 95% CI 19.3–24.7 months vs. 19.4 months, 95% CI 16.9–22.9 months, HR 1.099, 95% CI 0.958–1.26, *p* = 0.178). The same was true in those with pN2/LVI+ disease compared with pN3/LVI− where the median survival was 15 months (95% CI 13.2–16.8 months) versus 16.2 months, respectively (95% CI 13.7–19.5 months, HR 0.999, 95% CI 0.821–1.216, *p* = 0.995). The worst overall survival was observed in those found to be pN3/LVI+ with a median survival of 11.9 months (95% CI 10–13.5 months; Table 4; Fig. 4).

## Discussion

In this study, we utilized the NCDB to evaluate the utility of LVI as an independent prognostic factor in EAC. We found LVI to be associated with a marked reduction in overall and median survival in this population. Additionally, LVI was associated with a significant increase in the likelihood of positive tumor margins at the time of resection. In a nodal upstaging model, patients with LVI in pN1 and pN2 disease demonstrated similar survival to those of one higher pN stage.

Historically, prognostication and treatment strategy in esophageal cancer has been reliant on the well-established AJCC TNM staging protocol and associated NCCN guidelines. These guidelines were heavily influenced by the CROSS trial in 2012 with the establishment of neoadjuvant chemoradiation (CRT) followed by esophagectomy as the standard of care in those with T1b or greater disease.^7^ Following this standardization of therapy and recent advancements in targeted chemotherapeutic and immunologic regimens, much research has focused on prognostic factors in esophageal cancer. Lymphovascular invasion, defined as the presence of malignant cells in the endothelium of local lymphatics or blood vessels, has been identified as a strikingly poor prognostic factor in a variety of cancers, including both EAC and ESCC.^5,8^ The presence of LVI is largely accepted as a marker of either concomitant or imminent nodal involvement; however, it is not currently evaluated as a factor in pathologic staging. Previous studies have demonstrated similarly poor outcomes in those with LVI compared with their pN1 counterparts, offering LVI as an independent prognostic factor.^4,7,9^ Their findings were threefold: nodal disease was significantly associated with the presence of LVI (OR 1.52, 95% CI 1.353–1.708, *p* < 0.001), the presence of LVI significantly decreased overall survival in pN0, pN2, and pN3 disease, and the presence of LVI portended decreased overall survival as if upstaged to the next nodal stage. This becomes relevant when considering the standard of care established by the JCOG9204 trial in 2003 in which patients with nodal involvement receive adjuvant chemotherapy following esophagectomy.^10–12^ The potential utility of this trend in both pathologic staging and determination of adjuvant therapy raised the question: should patients with LVI in the absence of nodal disease receive adjuvant chemotherapy as if they were node-positive?^9,13^

To the best of our knowledge, current literature has only evaluated survival trends associated with LVI in ESCC. These data have established LVI as a strong independent prognostic factor with speculation of its utility as an adjunct to the pN staging system and potential indication for adjuvant treatment regimens.^4,13,14^ To address the paucity of data in EAC, we identified nearly 10,000 patients in the NCDB that received neoadjuvant therapy and underwent esophagectomy. Roughly a quarter of those patients were found to have LVI at the time of resection. We sought to identify factors predictive of LVI, evaluate the utility of LVI as an independent prognostic factor, and quantify the impact of LVI on survival trends.

### Prognostic Indicators of LVI

Although our primary focus was the utility of LVI as an independent prognostic factor for survival, we first evaluated specific patient and tumor characteristics associated with the presence of LVI at resection. The population of patients with LVI was largely similar to allcomers when considering both demographic and clinical characteristics (Table 1). The majority of patients included in the study were diagnosed with clinical stage III disease irrespective of LVI status, although those with LVI were significantly more likely to be diagnosed with advanced stage disease. This mirrors current trends in advanced-stage diagnosis of esophageal adenocarcinoma.^15^ Those with LVI were marginally younger with a minimal increase in time from diagnosis to surgery. They were more likely to receive treatment at an academic institution (Table 2).

Interestingly, on logistic regression modeling, we did not identify any socioeconomic factors associated with LVI. Those characteristics examined included sex, race, ethnicity, insurance payor, and income and education status. Increasing age (by 1 year) was associated with slightly reduced odds of LVI (OR 0.993, *p* = 0.0163). To address the common overlap in socioeconomic risk factors, our model adjusted for insurance payor and income and education status surrogates. Treatment facility was found to be persistently significant in this model, with those receiving care at comprehensive community cancer programs being slightly less likely to have LVI compared with an academic research facility (OR 0.846, *p* = 0.04). It is unclear whether these findings are true clinical trends or a product of variation in histologic evaluation between academic and community partners. With such a strong impact on survival, these data potentially underscore the need for accurate and standardized evaluation of LVI at resection to enable improved prognostication, adjuvant therapy, and surveillance across facility types.

Unsurprisingly, clinical stage was a strong predictor of LVI. Those with clinical stage II disease were notably more likely to have LVI at resection (OR 1.354, *p* < 0.0001), whereas those with clinical stage III disease had more than a two-fold increase in likelihood of LVI (OR 2.144, *p* < 0.0001). Similar trends have been demonstrated in ESCC.^6,9^ While accurate and complete evaluation of LVI is necessary in all patients, these data offer support for increased suspicion in those with advanced clinical disease.

### LVI as an Independent Prognostic Factor

Those patients with LVI demonstrated significantly reduced overall and median survival compared to their non-LVI counterparts. In allcomers, those with LVI had a median survival of just 20.0 months, a nearly 50% reduction from the median survival of 39.7 months in those without LVI. Four-year survival was also markedly affected with a reduction from 45.8% in those without LVI to just 26.4% in those with LVI (Fig. 2).

On multivariable analysis, LVI was found to be an independent prognostic factor for survival irrespective of pathologic stage (HR 1.401, *p* < 0.0001). Interestingly, LVI had a stronger negative impact than clinical stage III disease (HR 1.265, *p* < 0.001; Table 3). This is particularly striking as current NCCN guidelines consider only pathologic TNM staging in determination of adjuvant therapy regimen; LVI is not currently factored into this algorithm.

We also completed a subset analysis evaluating the association between LVI and positive margins on resection. Those with LVI demonstrated a nearly twofold increase in residual tumor following resection (HR 1.925, *p* < 0.0001). For comparison, LVI was a stronger predictor of a positive resection margin than pathologic stage T2 disease (HR 1.144) as well as N2 involvement (HR 1.679). Within our study cohort, a positive margin was associated an increased hazard of death (HR 1.466, *p* < 0.0001; Table 4). The reduction in survival conveyed by LVI persisted in those with residual margins; median survival in those with residual margins and LVI was just 10.8 months compared with 19.3 months in those with positive margins without LVI (Fig. 3).

### LVI and Nodal Status Upstaging

Because LVI often is viewed as a precursor to nodal involvement, we sought to evaluate the utility of LVI as a surrogate for upstaged nodal disease, particularly in the setting of such poor survival trends in those with LVI. Wang et al. demonstrated a similar model in ESCC in which pN0/LVI+ patients had comparable survival to those with pN1/LVI− disease. In their model, the same was true for pN1/LVI+ and pN2/LVI−, as well as pN2/LVI+ and pN3/LVI− disease.^6^

Following multivariable analysis adjusting for pathologic stage, our nodal upstaging model demonstrated similar findings to those detailed by Wang et al. in ESCC. The pN0/LVI+ population demonstrated a significantly reduced median survival compared with pN0/LVI− patients (34.3 months vs. 51.6 months, *p* = 0.0001) and a more similar survival to the higher staged pN1/LVI− patients, although not statistically equivalent (34.3 months vs. 28.6 months, *p* = 0.0135). No significant difference in median survival was found between pN1/LVI+ patients and pN2/LVI− patients (21.6 months vs. 19.4 months, *p* = 0.178). A similar trend was seen in those with pN2/LVI+ disease compared with pN3/LVI− where the median survival was 15 months vs. 16.2 months respectively (*p* = 0.995; Table 5; Fig. 4). This striking reduction in survival of LVI+ patients in a given pN stage underscores the impact of LVI on outcomes in EAC, particularly in those without overt nodal involvement.

**Table 5:** Kaplan–Meier and Subgroup Cox Proportional Hazard Overall Survival by pN and LVI Status

	Median survival (95% CI) (months)^1^	Four-year survival (95% CI)^1^	Subgroup adjusted hazard ratio (95% CI)^2^	p-value^2^
pN0 LVI-	51.6 (47.8–56.2)	51.6% (49.9–52.3%)		
pN0 LVI+	34.3 (28.4–40.1)	42.7% (38–4–47.4%)	Reference	**0.0135**
pN1 LVI-	28.6 (26.2–31.2)	35.6% (33.2–38.2%)	1.197 (1.038–1.380)	
pN1 LVI+	21.6 (19.3–24.7)	26.9% (23.5–30.7%)	Reference	0.1780
pN2 LVI-	19.4 (16.9–22.9)	24.6% (21.0–28.9%)	1.099 (0.958–1.260)	
pN2 LVI+	15.0 (13.2–16.8)	16.7% (13.7–20.5%)	Reference	0.9953
pN3 LVI-	16.2 (13.7–19.5)	15.1% (10.1–22.7%)	0.999 (0.821–1.216)	
pN3 LVI+	11.9 (10.0–13.5)	10.0% (6.6–15.1%)		

Bold value indicates statistically significant at *p* < 0.05

^1^Kaplan–Meier method

^2^Multiavariable Subgroup Cox models

Based on this analysis, we propose that LVI could serve as a valuable adjunct to the current pN staging system for EAC. Our data demonstrate statistically equivalent survival in those with pN1 and pN2 disease compared to those with a more advanced pN stage. This implies that those patients should be classified as such to reflect more accurately their prognosis. Additionally, although pN0 patients with LVI have slightly better survival than those with pN1 disease without LVI, their overall survival is substantially worse than their pN0/LVI− counterparts and would be better prognosticated as pN1 disease. While there have been previous studies supporting the incorporation of LVI into the pN staging system for various other cancers, including ESCC, to our knowledge, this is the first report to do so in EAC.^4,6^

This upstaging model carries implications both for better prognostication as well as candidacy for adjuvant therapy. Currently, patients with evidence of nodal disease at the time of resection are candidates for additional therapies following surgery.^2^ Our data elucidate an important question: given that pN0 patients with LVI have significantly worse survival than those without LVI and their survival much more closely reflects those with nodal involvement, should these patients be candidates for adjuvant therapy? Future studies with consideration of a randomized controlled trial evaluating the impact of adjuvant therapy in this subset of patients is warranted. Additionally, recent conversation surrounding the benefit of extended lymphadenectomy in EAC extends to this patient group. Because LVI often is considered a precursor to true nodal involvement, it is feasible that the reduction in survival seen in those with LVI may in part be driven by limited lymph node harvest failing to capture the presence of positive nodes, thus resulting in pathologic understaging and inaccurate prognostication. Further evaluation of LVI as a high-risk factor for unidentified nodal involvement and the associated impact on survival is warranted.

### Limitations

We recognize that the present study is not without limitations. The NCDB is a large-scale database that only includes programs accredited by the Commission on Cancer (CoC). Despite the large patient population and thus high level of statistical power, the potential for selection bias, lack of granular disease-specific and socioeconomic data, and significant variation in coding practices limits generalizability. Additionally, the NCDB does not capture data regarding disease recurrence and offers only limited detail regarding surgical findings. We also note that specific information regarding neoadjuvant regimen is not available in the NCDB. Continued study in the modern era will be important as treatments continue to evolve rapidly.

## Conclusions

The utility of LVI as an independent predictor of outcomes in EAC is quite pronounced, particularly when evaluated in the absence of nodal involvement. Our data suggest that those with LVI demonstrate similar outcomes to the next most advanced nodal stage. With additional investigation, there is potential for LVI to serve a significant role in staging and thus prognostication and determination of adjuvant therapy regimen.
